# Human Papilloma Virus Infection and Sinonasal Inverted Papilloma Recurrence: A Meta‐Analysis

**DOI:** 10.1002/ohn.1108

**Published:** 2024-12-31

**Authors:** Fayssal Alqudrah, Sharwani Kota, Jason Morgan, Phillip R. Purnell, Justin P. McCormick

**Affiliations:** ^1^ Department of Otolaryngology–Head and Neck Surgery Rutgers Robert Wood Johnson Medical School New Brunswick New Jersey USA

**Keywords:** human papillomavirus, inverted papilloma, meta‐analysis, recurrence, sinonasal tumor

## Abstract

**Objective:**

Prior studies have been contradictory on the role of human papillomavirus (HPV) infection in sinonasal inverted papilloma (SNIP) recurrence. This systematic review and meta‐analysis was performed to further evaluate this potential association.

**Data Sources:**

PubMed, Embase, and Scopus electronic databases.

**Review Methods:**

Case‐control studies reporting SNIP recurrence data and HPV status identified by polymerase chain reaction (PCR) and in‐situ hybridization (ISH). Meta‐analysis was performed to determine pooled odds ratios (ORs) and 95% confidence intervals (CI).

**Results:**

25 studies were identified including a total of 1116 benign SNIP tumors. A total of 267 SNIP were HPV+, 103 of which were recurrent, and 849 SNIP were HPV−, with 231 being recurrent. The pooled standard OR for recurrence in HPV+ tumors was 2.05 (95% CI: 1.31‐3.19). Stratification by low‐risk and high‐risk HPV subtypes were not statistically significant. The standard OR for SNIP recurrence in low‐risk and high‐risk HPV+ subtypes were 1.57 (95% CI: 0.98‐2.54) and 1.67 (95% CI: 0.98‐2.80), respectively.

**Conclusion:**

Infection with HPV may be associated with an increased risk of SNIP recurrence. This increased risk seems to be independent of HPV subtype based on low‐risk or high‐risk status. However, this correlation was variable among recently published studies requiring additional investigation.

Sinonasal inverted papillomas (SNIP) are benign epithelial tumors that arise from the sinonasal cavity and represent up to 5% of primary sinonasal neoplasms.^1^, ^2^ The exact etiology of SNIP remains unclear, however, smoking, chronic inflammation, angiogenic proteins, and industrial exposures have been reported to potentially play a role in the development of SNIP.^3^ Several studies have also investigated viral mediated pathogenesis, with particular focus on Epstein‐Barr virus (EBV) and human papillomavirus (HPV).^4^, ^5^, ^6^

HPV is a DNA virus that can drive oncogenesis through insertion of the viral oncogenes, E6 and E7, into their host cell DNA. HPV subtypes are divided into high‐ and low‐risk categories based on their potential for oncogenesis (Table 1). With differences in HPV detection methods, results can be inconsistent with varying interpretations across studies.^7^ A prior study identified a preponderance of low‐risk HPV in benign SNIP, and reported an odds ratio (OR) of 10.2 for recurrence in HPV+SNIP compared to HPV− SNIP.^4^ Additionally, a recent large case series identified significantly higher HPV positivity in recurrent SNIP compared to non‐recurrent SNIP.^8^ However, other studies have suggested that HPV in SNIP may just represent colonization as opposed to being a true etiological factor.^9^, ^10^ Despite these suggestions, recent meta‐analyses indicate that the presence of HPV may have an impact on the clinical course of SNIP in relation to recurrence, and even malignant transformation.^9^, ^11^

**Table 1:** HPV Subtypes Categories

High‐risk	Low‐risk
HPV‐16, ‐18, ‐31, ‐33, ‐34, ‐35, ‐39, ‐45, ‐51, ‐52, ‐56, and ‐58	HPV‐6, ‐11, ‐42, ‐43, and ‐44

Specific HPV subtypes based on high‐ and low‐risk potential for HPV oncogenesis.

This meta‐analysis seeks to expand on prior work in identifying the role of HPV in SNIP recurrence. Additionally, the independent role of high‐ and low‐risk HPV subtypes was investigated. PICOS criteria along with a description of the quality assessment tool used can be found in the Supplemental File.

## Methods

### Literature Search

A systematic review of literature was conducted adhering to the PRISMA statement and performed by searching PubMed, EMBASE, and SCOPUS databases from inception up to February 5, 2024. The search strategy employed entailed: (“inverted papilloma” OR “sinus papilloma” OR “sinonasal papilloma” OR “sinus tumor”) AND (“human papillomavirus” OR “papillomavirus” OR “HPV”) AND (“sinus” OR “sinonasal” OR “nasal” OR “nose”). Filters were subsequently applied to only include English language‐based articles. References of the included articles were also reviewed to find additional relevant publications.

### Study Selection

Articles included within the study were case‐control, prospective and retrospective cohort studies to investigate the association between HPV status and recurrence rate of SNIP. While there is no gold‐standard for detection of HPV in tissues, we chose to include only articles using polymerase chain reaction (PCR) and in‐situ hybridization (ISH) based on their high sensitivity and frequent utilization for HPV detection.^11^, ^12^ In the main analysis, both high‐risk HPV (16, 18, 31, 33, 34, 35, 39, 45, 51, 52, 56, 58) and low‐risk HPV (6, 11, 42, 43, and 44) subtypes were included under the HPV positive category. Articles were excluded if: the primary tumor was non‐SNIP, HPV DNA was detected by any means other than PCR and ISH, or recurrent tumor data was not included.

### Data Extraction

Extracted data were reviewed and agreed upon by all 4 authors with any discrepancies resolved by majority vote. Extracted information included the name of the first author, publication year, average years in age, sex, follow‐up duration, HPV detection method (PCR/ISH), country, number of SNIP, HPV status and subtype, and associated recurrence rate with low/high‐risk HPV positivity.

### Statistical Analysis

Using summary data from each of the individual studies, odds ratios (ORs) and 95% confidence intervals (CIs) were calculated. For ORs, 0.5 was added to each cell if any one of the cells included zero based off of the Haldane‐Anscombe correction for OR. Using the random‐effects model in Stata software (StataCorp 2019) pooled log‐ORs were generated and subsequently converted to standard OR. Study heterogeneity was then evaluated using *I* ^2^ and Cochran *Q* statistic. Two subgroup analyses for recurrence rate were performed within the high‐risk and low‐risk HPV tumor populations. Within the high‐risk subgroup analysis, tumors which were positive for low‐risk HPV were included in the HPV‐negative group. Similarly, within the low‐risk subgroup analysis, tumors which were positive for high‐risk HPV were included in the HPV‐negative group.

### Risk of Bias Assessment

The Newcastle‐Ottawa Scale (NOS) for assessment of the quality of nonrandomized studies was used to assess for risk of bias within the studies.

## Results

**Figure 1:**
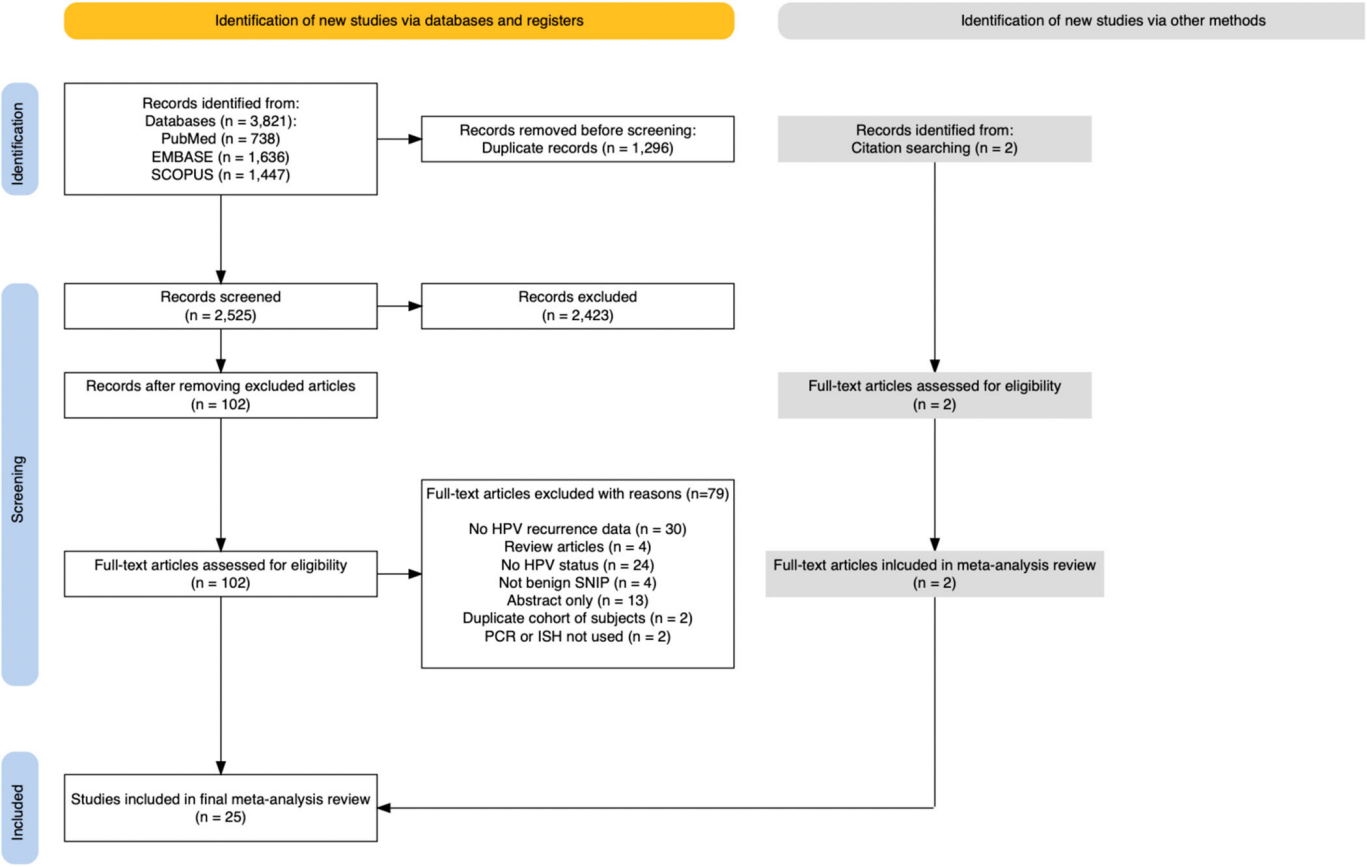
Meta‐analysis study selection flowchart diagram.^13^

A total of 3821 articles were retrieved from the initial database search, of which 1296 were duplicate records. After initial review of titles and abstracts, 2423 articles were additionally excluded. One hundred and two articles were reviewed in full and 2 additional titles were included after screening references of the reviewed articles. Of the 104 articles undergoing full‐text review, 79 were excluded. Of the 79 excluded articles, 30 lacked HPV recurrence data, 4 were review articles, 24 did not indicate the HPV status, 4 included only malignant SNIP tumors, 13 were only abstracts, 2 had duplicate cohort of study subjects, and 2 did not utilize PCR or ISH. This led to the final inclusion of 25 studies in this meta‐analysis study. The study selection process is illustrated in Figure 1.

The included studies were published between January 1, 1988 and August 11, 2023, and study characteristics are listed in Table 2. Sample size of the studies ranged from 9 to 109 subjects. A total of 1116 benign SNIP tumors were included in the analysis. Of the total identified benign SNIP tumors, a total of 267 SNIP tumors tested positive for HPV infections, with 103 identified as recurrent and 164 as nonrecurrent. The remaining 849 SNIP tumors tested negative for HPV infection, with 231 identified as recurrent and 618 as nonrecurrent. There were 140 tumors that were low‐risk HPV positive, of which 43 were recurrent and 97 were nonrecurrent. One‐hundred and four tumors were high‐risk HPV positive, of which 36 were recurrent and 68 were nonrecurrent. There were a total of 242 tumors, 47 positive for HPV and 195 negative for HPV, that did not report the HPV subtype.

**Table 2:** Characteristics of Selected Studies

Author	Date	Country	Follow‐up duration (months)	HPV detection method	NOS	Total subjects, n	HPV+		HPV−	
							Rec, n (%)	No Rec, n (%)	Rec, n (%)	No Rec, n (%)
Weber^14^	1988	USA	40.4	ISH	6	21	7	9	0	5
Furuta^15^	1991	Japan	NR	PCR & ISH	5	21	2	2	4	13
Beck^16^	1995	USA	40^a^	PCR	6	22	10	2	0	10
Ogura^17^	1996	Japan	NR	PCR	5	9	2	1	1	5
Bernauer^18^	1997	Germany	37.6	PCR	6	21	2	5	1	13
Hwang^19^	1998	Korea	24	PCR	6	36	2	1	2	31
Kraft^20^	2001	Switzerland	NR	PCR & ISH	5	25	0	1	5	19
Jenko^10^	2011	Slovenia	NR	PCR	5	66	5	15	9	37
Hasegawa^21^	2012	Japan	17^a^	PCR	6	13	1	5	1	6
Giotakis^22^	2012	Greece	48	PCR	6	32	4	6	5	17
Scheel^23^	2015	USA	51.8^a^	PCR	6	86	7	4	34	41
Jung‐Roh^24^	2016	Korea	40.6	PCR	6	54	0	8	7	39
Lin^25^	2016	China	NR	PCR	5	28	12	3	4	9
Elliot^26^	2019	Sweden	60^a^	PCR	5	98	4	8	36	50
Fulla^9^	2020	Spain/Poland	52.83^a^	PCR	6	76	1	3	16	56
Holm^27^	2020	Sweden	3.5	PCR	5	38	1	1	19	17
Cabal^28^	2020	Spain	141	PCR	5	40	0	4	16	20
Frasson^29^	2020	Italy	35^a^	PCR	6	55	9	25	7	14
Husain^30^	2020	Malaysia	NR	PCR	5	41	2	11	5	23
Paehler vor der Holte^8^	2021	Germany	NR	PCR	6	109	16	31	13	49
Viitasalo^31^	2021	Finland	36.1^a^	PCR	6	90	13	5	25	47
Menendez^32^	2022	Spain	NR	PCR	6	36	0	2	16	18
Tsumura^33^	2022	Japan	57.3	PCR	5	32	3	2	4	23
Hirakawa^34^	2023	Japan	24^a^	PCR	6	20	0	5	1	14
Hu^35^	2023	China	22.9	PCR	6	47	0	5	0	42

Individual study characteristics reporting on SNIP recurrence rate based on high‐ and low‐risk HPV subtypes.

Abbreviations: HPV, human papillomavirus; ISH, in‐situ hybridization; NOS, Newcastle Ottawa scale; NR, not reported; PCR, polymerase chain reaction.

^a^ Median.

**Figure 2:**
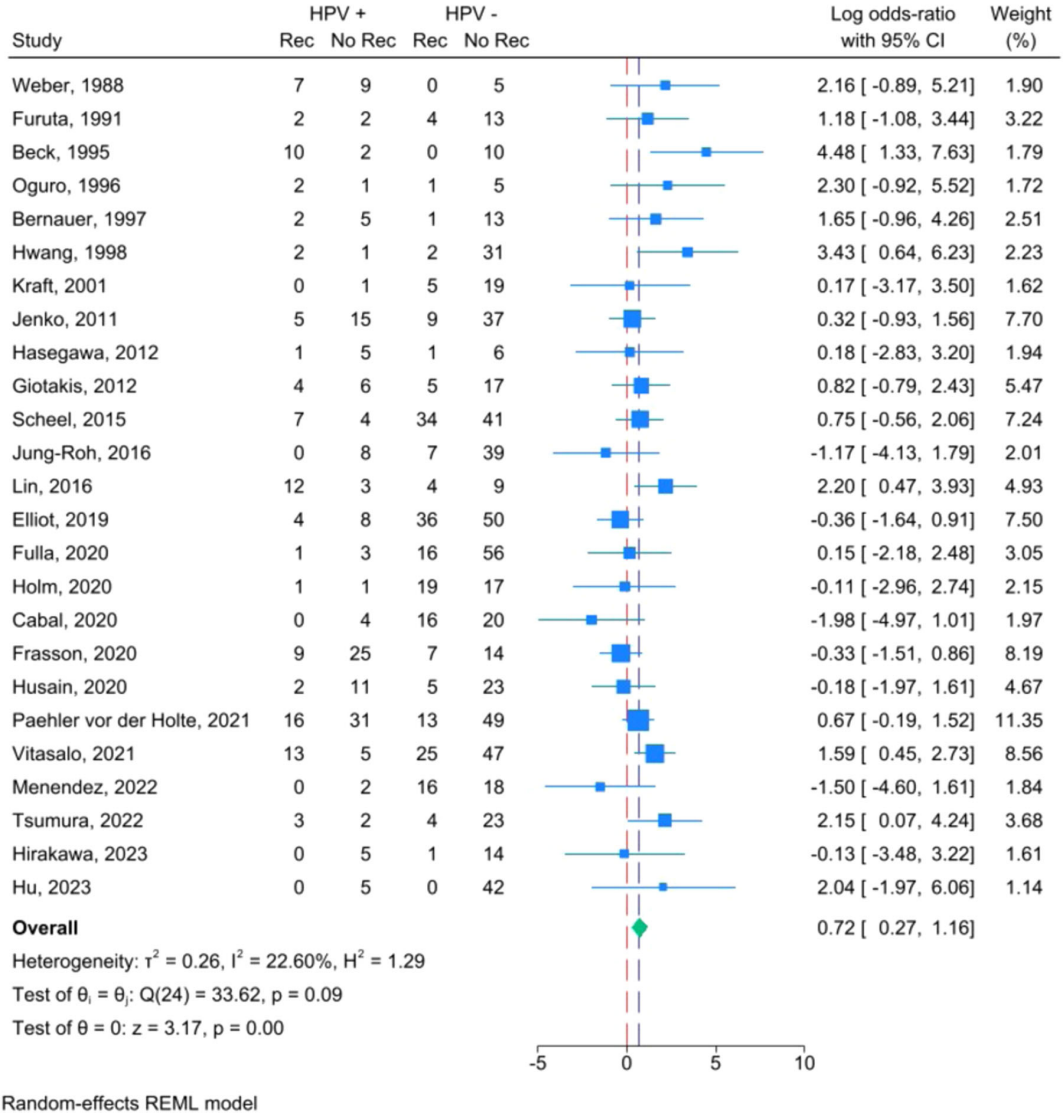
Forest plot including all HPV subtypes tumor recurrences as being positive for HPV. Effect size is listed as log‐OR. No rec, nonrecurrent tumor; Rec, recurrent tumor.

**Figure 3:**
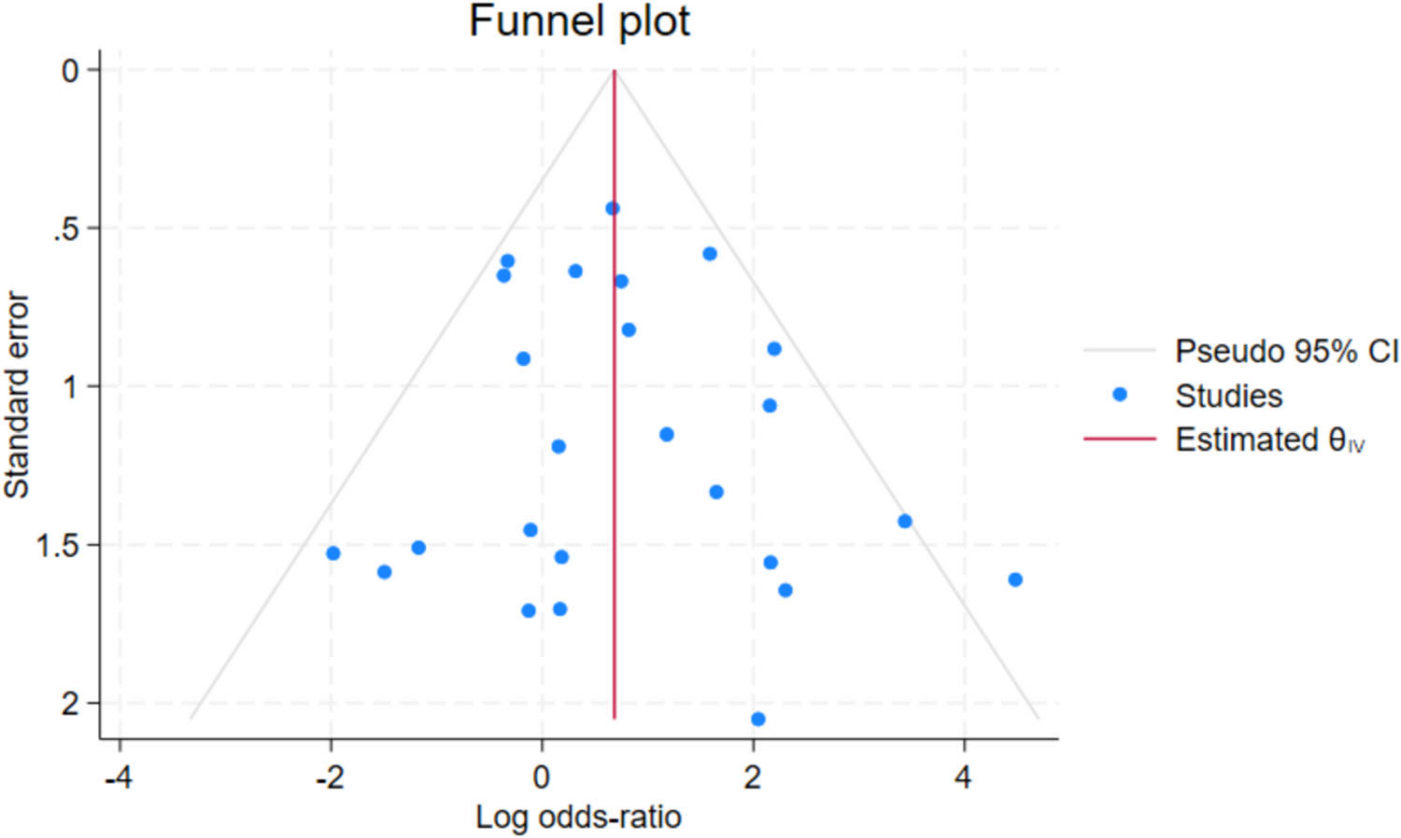
Funnel plot with Egger test performed to assess for small‐study effect on publication bias.

The weighted average effect size including all HPV+ subtypes on tumor recurrence was log‐OR: 0.72; 95% CI: 0.27 to 1.16 (Figure 2). This converts to a standard OR of 2.05; 95% CI: 1.31 to 3.19. Overall there was low dispersion of effect sizes with *I* ^2^ = 22.60% and *τ* ^2^ = 0.26. The associated funnel plot (Figure 3) was visually symmetric. The Egger test for small‐study effect suggested no significant evidence of publication bias (*t* = 0.67; *p* = 0.5075).

**Figure 4:**
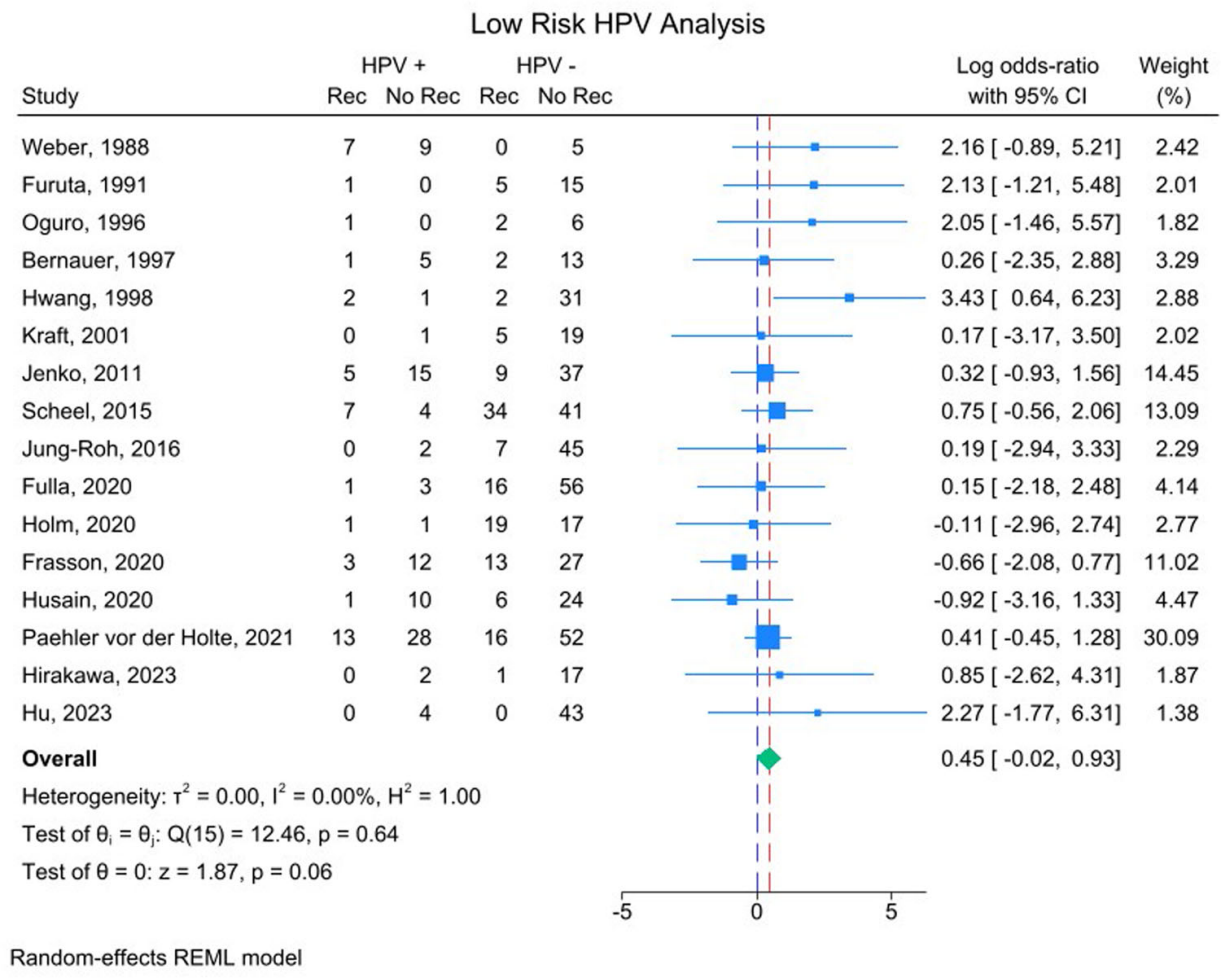
Forest plot including only recurrences in tumors positive for low‐risk HPV subtypes as being positive for HPV. Effect size listed as log‐OR. No rec, nonrecurrent tumor; Rec, recurrent tumor.

**Figure 5:**
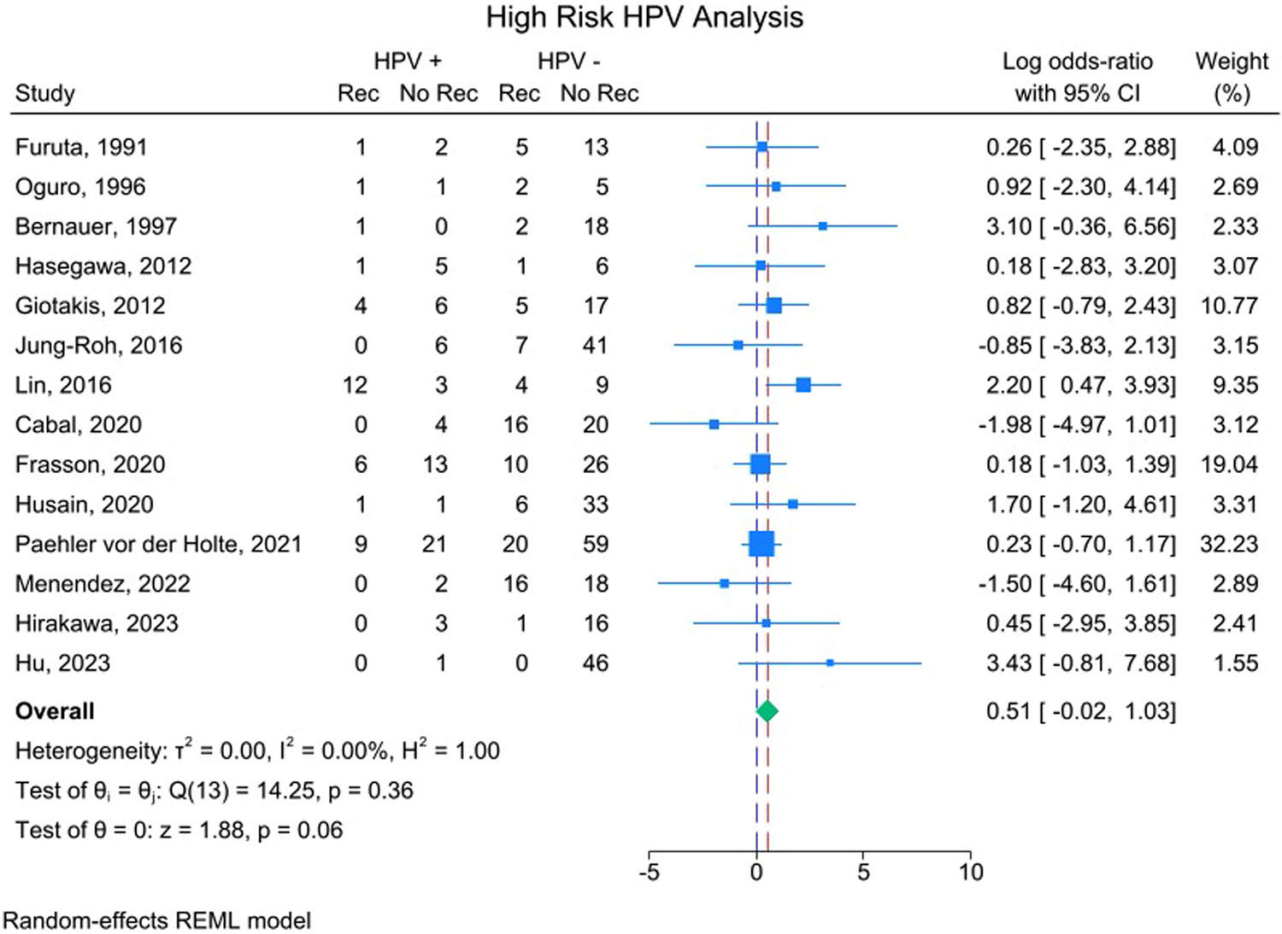
Forest plot including only recurrences in tumors positive for high‐risk HPV subtypes as being positive for HPV. Effect size listed as log‐OR. No rec, nonrecurrent tumor; Rec, recurrent tumor.

Separate meta‐analyses were then performed including only studies that differentiated tumors based on low‐ and high‐risk HPV subtypes. Sixteen studies analyzed recurrences in tumors positive for low‐risk HPV subtypes, but no statistically significant association was found for tumor recurrence (*P* > .05). The weighted average effect size including only tumor recurrences positive for low‐risk HPV subtypes was log‐OR: 0.45; 95% CI: −0.02 to 0.93 (Figure 4). This converts to a standard OR of 1.57; 95% CI: 0.98 to 2.54. There was no significant dispersion of effect sizes, *I* ^2^ = 0.00% and *τ* ^2^ = 0.00. Fourteen studies analyzed recurrences in tumors positive for high‐risk HPV subtypes, but no statistically significant association was found for tumor recurrence (*P* > .05). The weighted average effect size of this analysis was log‐OR: 0.51; 95% CI: −0.02 to 1.03 (Figure 5). This converts to a standard OR of 1.67; 95% CI: 0.98 to 2.80. Dispersion of effect sizes was also not significant with *I* ^2^ = 0.00% and *τ* ^2^ = 0.00.

A subgroup analysis based on date of publication within and prior to the last decade (year 2014) was also performed. There was no statistically significant association of HPV positivity and tumor recurrences from 2014 to the present day, log‐OR: 0.45; 95% CI: −0.11 to 1.01, which is equivalent to standard OR of 1.57; 95% CI: 0.90 to 2.75. There was, however, a statistically significant association of HPV positivity and tumor recurrence prior to the last decade, log‐OR: 1.31; 95% CI: 0.50 to 2.12 which is equivalent to standard OR of 3.71; 95% CI: 1.56 to 8.38.

## Discussion

The investigation into the relationship of HPV prevalence in SNIP tumors has evolved since it was initially explored, over 35 years ago, by Syrjanen et al.^36^ While several new studies explored the pathogenesis of SNIP tumors in relation to HPV infections, the results remain inconclusive regarding the exact role of HPV in the development, potential for malignant transformation, and recurrences of SNIP tumors.^8^, ^37^, ^38^ Additionally, certain HPV subtypes such as HPV‐16 and HPV‐18 carry higher oncogenic risk accounting about 85% of HPV‐related head and neck cancers.^39^ In a recent study by Tong et al, the prevalence of oncogenic HPV subtypes was studied across 961 benign SNIP tumors and 163 malignant SNIP tumors. The study showed the prevalence of oncogenic HPV subtypes was 14% in benign SNIP tumors and 73% in SNIP‐associated squamous cell carcinoma tumors.^40^ A study by Wang et al investigated the presence of high‐risk HPV, HPV‐16 & ‐18, in patients with benign SNIP, SNIP with dysplasia, and malignant SNIP. The study detected high‐risk HPV in 3.1% of SNIP and SNIP with dysplasia tumors cumulatively compared to 42.9% of malignant SNIP tumors.^41^ In a previous meta‐analysis, the association of HPV‐16 and HPV‐18 after stratification by high‐risk HPV subtype was found to be correlated with a 5.31 and 14.59 higher odds of malignancy, respectively.^5^ With these valuable insights into the potential role of HPV in malignant transformation of SNIP, we wanted to further explore the potential role of HPV in recurrence of benign SNIP.

Previous studies have investigated the relationship between the presence of HPV infection and SNIP tumor recurrences. Lawson et al demonstrated a significant association between HPV+ and SNIP recurrences. The weighted prevalence of HPV+ in recurrent benign SNIP tumors was 57.9% compared to 9.7% among nonrecurrent benign SNIP tumors.^4^ Additionally, Pahler vor der Holte and colleagues reported 64.3% of all recurrent SNIP tumors were positive for HPV.^8^ A recent meta‐analysis by Rha et al combined case studies evaluating the association between HPV positivity and SNIP recurrence through 2021. The final analysis of 14 studies published between 1988 and 2021, concluded that HPV positivity was significantly linked to an increased risk of SNIP recurrence.^42^ These findings suggest that HPV infections may play a role in the recurrence of SNIP tumors.

While emerging data from recent studies have supported HPV infections being a causative risk factor rather than a coincidental colonization, the exact pathogenesis and association between HPV infections and SNIP remains poorly understood. The question remains, however, whether HPV positivity of SNIP should change management. Given that the current meta‐analysis and multiple other recent studies have shown a correlation between HPV and SNIP recurrence, surgeons could consider more aggressive resection or wider margins at the initial surgery for HPV+ tumors. Additionally, increasing the frequency of surveillance visits and lowering the threshold for biopsy of any recurrent lesion in HPV+ tumors could be considered. Lastly, although there is no clear evidence supporting the administration of the HPV vaccination in patients with current HPV‐related disease, physicians can discuss with patients that vaccination may prevent infection from new subtypes or re‐infection from autoinoculation from an adjacent site, although how this will affect SNIP outcomes will need further study.^43^

This current systematic review and meta‐analysis is distinct from that of Rha and colleagues, as 11 additional studies were identified, while further evaluating the independent effects of both high‐risk and low‐risk HPV subtypes on SNIP recurrence. The pooled results of this study suggest that patients with HPV + SNIP have a significantly higher likelihood of having tumor recurrence compared to HPV‐negative specimens. Interestingly, however, the separate analysis based on HPV subtype is not statistically significant. While this highlights the ongoing uncertainty about the impact of HPV on SNIP recurrence, this finding could have been impacted by co‐infection of some tumors with both high‐ and low‐risk subtypes, which was not always well documented in the included studies. Additionally, not all of the initial 25 studies included subtype data, and thus were not able to be included in the separate meta‐analyses. This study helps emphasize the importance of future research to understand the implications of HPV on inverted papilloma recurrence.

Given the wide variation of date ranges of the included studies, we also wanted to investigate whether there was a difference in tumor recurrence based on date of publication. We arbitrarily chose to include studies published within the last decade in the more recent cohort, and those published prior to 2014 in the older cohort. This subgroup analysis revealed a significant association with HPV+ tumors and recurrence rate in those studies published prior to 2014, but no significant association in those studies published more recently. This can potentially be attributed to a better understanding of inverted papilloma treatment, improved surgical visualization, and advanced surgical approaches allowing improved access for complete tumor extirpation. Historically, open approaches were utilized for SNIP excision. Advancements in minimally invasive surgical techniques, along with increased surgeon experience, have improved visualization allowing for more complete resection of SNIP tumors.^44^ A review article published in 2019 reported the recurrence rate of SNIP to be about 36.7% in the traditional open group compared to 13.5% in the endoscopic group. In a recent meta‐analysis by Peng et al, the endoscopic approach group had a significant 39% lower risk of SNIP recurrence rate compared to the open approach group.^45^ These surgical factors may have contributed to the lower recurrence rates observed in the more recent studies. An additional consideration which could be investigated further is whether the introduction of the HPV vaccine has any effect on inverted papilloma incidence, recurrence, or malignant transformation.

This systematic review, like all meta‐analyses, has limitations to note. All articles chosen within the final full‐text screening were screened for English language only, which could exclude articles of significance due to language bias. Moreover, many of the articles which were included in the overall analysis did not include HPV subtype data. We did attempt to contact authors to obtain subtype data, however, for many we were not able to receive this information. As such, this could introduce bias into the sub‐group analyses differentiating recurrence based on high‐risk and low‐risk HPV status. Finally, while standard systematic review guidelines were followed, the possibility of missed publications during our review process is also considered.

## Conclusion

The findings of this meta‐analysis suggest that HPV positivity may contribute to SNIP recurrence. However, the variability in correlation among recently published studies highlights the need for more prospective research studies to establish a definitive association between HPV subtypes and SNIP recurrence. Additionally, investigating the role of HPV vaccination on inverted papilloma incidence, recurrence, and malignant transformation may further clarify the role of HPV in this disease process.

## Author Contributions

**Justin P. McCormick**, study idea, design, conduct, interpretation, analysis, manuscript drafting, critical manuscript revision, final approval for publication; **Fayssal Alqudrah**, study design, conduct, interpretation, manuscript drafting, revision, and manuscript final approval of publication; **Sharwani Kota**, study conduct, interpretation, manuscript drafting and revision; **Jason Morgan**, study conduct, interpretation, and manuscript drafting; **Phillip R. Purnell**, data analysis and interpretation, and manuscript revision for intellectual content.

## Disclosures

### Competing interests

None.

### Funding source

None.
